# Plasma Membrane Fluidity: An Environment Thermal Detector in Plants

**DOI:** 10.3390/cells10102778

**Published:** 2021-10-17

**Authors:** Dora L. Cano-Ramirez, Laura Carmona-Salazar, Francisco Morales-Cedillo, Jorge Ramírez-Salcedo, Edgar B. Cahoon, Marina Gavilanes-Ruíz

**Affiliations:** 1 Departamento de Bioquímica, Conjunto E, Facultad de Química, Universidad Nacional Autónoma de México, UNAM, Cd. Universitaria, Coyoacán, Ciudad de México 04510, Mexico; dlc55@cam.ac.uk (D.L.C.-R.); carmonal@comunidad.unam.mx (L.C.-S.); fran_0988@hotmail.com (F.M.-C.); 2 Unidad de Microarreglos, Instituto de Fisiología Celular, Universidad Nacional Autónoma de México, UNAM, Cd. Universitaria, Coyoacán, Ciudad de México 04510, Mexico; jramirez@ifc.unam.mx; 3 Center for Plant Science Innovation & Department of Biochemistry, University of Nebraska-Lincoln, Lincoln, NE 68588-0665, USA; ecahoon2@unlnotes.unl.edu

**Keywords:** lipids, membrane fluidity, membrane lipids, plasma membrane, plant membranes, temperature perception

## Abstract

The lipid matrix in cell membranes is a dynamic, bidimensional array of amphipathic molecules exhibiting mesomorphism, which contributes to the membrane fluidity changes in response to temperature fluctuation. As sessile organisms, plants must rapidly and accurately respond to environmental thermal variations. However, mechanisms underlying temperature perception in plants are poorly understood. We studied the thermal plasticity of membrane fluidity using three fluorescent probes across a temperature range of −5 to 41 °C in isolated microsomal fraction (MF), vacuolar membrane (VM), and plasma membrane (PM) vesicles from Arabidopsis plants. Results showed that PM were highly fluid and exhibited more phase transitions and hysteresis, while VM and MF lacked such attributes. These findings suggest that PM is an important cell hub with the capacity to rapidly undergo fluidity modifications in response to small changes of temperatures in ranges spanning those experienced in natural habitats. PM fluidity behaves as an ideal temperature detector: it is always present, covers the whole cell, responds quickly and with sensitivity to temperature variations, functions with a cell free-energy cost, and it is physically connected with potential thermal signal transducers to elicit a cell response. It is an optimal alternative for temperature detection selected for the plant kingdom.

## 1. Introduction

Cell membranes have a lipid matrix that behaves as a bi-dimensional organized solution in which lipid molecules exhibit the intrinsic property of mesomorphism. This is conferred by the amphipathic nature of lipids and their weak intermolecular interactions. Mesomorphism arises from the ability of lipid molecules to adopt different conformations in a temperature-dependent manner, thereby producing discrete physical states with specific properties [1,2,3]. Thus, low temperatures induce stiff, ordered, and packed arrays of lipids that promote membrane rigidity. Conversely, high temperatures promote flexible, disordered, and unpacked lipid conformations that result in a more fluid membrane [4,5]. A fluid state facilitates lateral movement of lipids, adequate conformational changes of integral proteins, and protein–protein and protein–lipid interactions [6]. These phenomena are vital for solute transport, signal transduction, cell communication, cell division, and many other cellular processes.

Homeotherms can keep their temperature constant and membrane fluidity is accordingly stable [7]. In contrast, poikilotherms, such as plants, lack mechanisms to regulate their temperature throughout the day/night cycle or across seasons. In this scenario, plants must rely on their ability to sense temperature changes and to respond accordingly by engaging appropriate short- and/or long-term physiological responses to preserve their functional integrity and survival. Therefore, it is reasonable to expect that plants possess mechanisms to perceive, regulate, and maintain the fluidity of their membranes at adequate levels across a range of temperatures.

The plant plasma membrane (PM) is a dynamic structure that can show sensitivity to temperature changes as a consequence of its lipid biophysical properties [8,9]. Early heat stress responses, such as Ca^2+^ influx to the cytoplasm, originate from channels and enzymes residing in the plasma membrane, which are thought to undergo conformational changes in response to membrane dynamics [10]. Hence, membrane lipid mesomorphism acquires relevance in plants, allowing any lipid bilayer to behave as a natural thermometer, albeit this mechanism has not yet been experimentally demonstrated in plant membranes [11]. An immediate change in lipid structure driven by sudden changes in environmental conditions can contribute to an instantaneous modification of the bilayer fluidity and membrane function. Changes in membrane viscosity may also unchain signaling events, leading to a longer-term, whole-cell adaptation in response to environmental temperature changes, such as synthesis of protectant proteins and lipids [8,12,13]. 

Current understanding of the lipid behavior of the PM under temperature variation is based on analyses of lipids extracted from whole plants or organs that have usually been exposed to long periods at low temperatures [14,15,16,17,18,19] or to short intervals at high temperatures [20,21]. 

This work aimed to study the dynamics of PM fluidity within a thermal range likely to occur under natural ambient conditions. To accomplish this, different plant vesicles were isolated and exposed to subtle and gradual variations in temperature and membrane fluidity was determined by fluorescence polarization (FP) of three reporter fluorescent probes. Results show that, in contrast to vacuolar membrane (VM) and microsomal fraction (MF), PM fluidity is highly sensitive to changes in temperature, responding fast and independently of cellular metabolism.

## 2. Materials and Methods

### 2.1. Plant Growth Conditions 

*Arabidopsis thaliana* (L.) seeds were germinated and grown in pots containing soil supplemented with agrolite and vermiculite (2:1:0.5, w:w:w). After three weeks, plantlets were transferred to fresh pots and grown in a greenhouse (22 °C with 8 h light and 16 h dark) for five weeks. Plants were irrigated with tap water during the first three weeks and with Hoagland solution once a week thereafter. Leaves were then harvested to isolate either MF, VM, or PM vesicles. 

### 2.2. Membrane Isolation

MF were obtained from homogenized leaves as described [22]. PM was purified from MF and separated by partitioning in two-phase aqueous polymer systems using dextran 500 (Sigma-Aldrich, St. Louis, MO, USA) and polyethylene glycol 3350 (Sigma-Aldrich, St. Louis, MO, USA) as described in [22]

VM or tonoplast were purified by fractionation of MF by free flow zonal electrophoresis (FFZE) using the BD FFE system (BD Proteomics, Munich, Germany), as described [23]. 

### 2.3. Measurement of Membrane Fluidity

Membrane fluidity was measured by fluorescence polarization (FP) using three fluorescent probes: 1,6 diphenyl-1, 3, 5-hexatriene (DPH, Sigma-Aldrich, St. Louis, MO, USA), 1-(4-trimethylammoniumphenyl)-6-phenyl-1,3,5-hexatriene *p*-toluenesulfonate (TMA-DPH, Sigma-Aldrich, St. Louis, MO, USA), and *cis*-parinaric acid (PA, Cayman Chemical, Ann Arbor, MI, USA) at excitation and emission wavelengths, respectively, of 340 nm/417 nm for DPH and TMA-DPH, and 320 nm/420 nm for PA.

DPH and TMA-DPH were dissolved in absolute dimethylformamide to a final concentration of 1 mM. PA was dissolved in butylhydroxytoluene and degassed ethanol (10 µg/mL) in the presence of nitrogen gas to a final concentration of 1 mM. Membrane preparations (300 µg of protein) isolated from *Arabidopsis thaliana* leaves were added to a solution of 620 mM sorbitol, 5 mM KH_2_PO_4_ (pH 7,8), 0.1 mM EDTA, and the appropriate fluorescent probe (1 µM for DPH and TMA-DPH, and 2 µM for PA) up to a final volume of 2.0 mL. The sample was then incubated under agitation at 1 °C for 15 min before measuring fluidity. Steady-state FP was recorded from −5 to 41 °C and back (41 to −5 °C), in 2 °C successive steps using a Peltier chamber. Steady-state FP values were determined in a SLM-AMINCO 48000 spectrofluorometer (SLM Instruments Inc., Urbana, IL, USA) equipped with light polarizers.

To measure polarization by our setting, light intensity emitted in the horizontal and vertical plane was recorded 6 times each and averaged to get one reading. This accounted for a technical replicate from six with an average standard deviation of 0.0025, even when some measurements were repeated after a 2-year gap. Therefore, for example, in VMs with a *n* = 2 based on independent membrane samples, each coming from over 70 plants, we had 6 technical replicates per timepoint (21 temperature points) in each direction (forward and reverse), which is summarized in a curve composed of over 252 determinations for VM with just one probe. In the case of the fluorescence polarization measurements for the MF and PM, the number of experimental data increased, since the number of independent membrane preparations were 4 and 6, respectively, and the technical replicates followed the same design described in the above paragraph for the VM.

### 2.4. Electron Microscopy

Transmission electron microscopy analysis of the membrane preparations was performed as described [22]. Ultrathin sections of 80 nm were observed with a Jeol 1200 EXII electron microscope (JEOL Ltd, Tokyo, Japan) operated at 60 kV.

### 2.5. Protein Determination

Protein concentrations were determined with a modified Lowry method using BSA (Sigma-Aldrich, St. Louis, MO, USA) as standard [24].

### 2.6. Immunoblotting

Membrane protein separation was performed by SDS-PAGE (10% acrylamide gel) according to Schägger and von Jagow [25]. Proteins were stained with Coomassie Blue (Sigma-Aldrich Corp. St. Louis, MO, USA) or electroblotted to PVDF membranes (Immobilon-P, Millipore Corp. Bedford, MA, USA) at 22 V for 2.5 h. These membranes were treated with Western Blot Signal Enhancer (Pierce^®^, Thermo Scientific, IL, USA) and blocked in 20 mM Tris, 150 mM sodium chloride pH 7.5 with 0.1% (*v*/*v*) Tween-20 (TBS-T) buffer with 2% defatted milk, and then successively incubated with the primary antibody and the second antibody. Antibody reacting bands were detected using alkaline phosphatase reaction (1:2500, Sigma-Aldrich, St. Louis, MO, USA) or anti-rabbit IgG horse radish peroxidase conjugated (1:20,000, Sigma-Aldrich, St. Louis, MO, USA). Antibodies used for immunoblotting were as follows: anti PIP2;1, PIP2;2, PIP2;3 (1:1000, Agrisera, Vännäs, Sweden, AS09 491), anti-Na^+^/H^+^ exchanger 1 (1:1000, Agrisera, Vännäs, Sweden, AS09 484), anti-SMT1 (1:1000, Agrisera, Vännäs, Sweden, AS07 266), anti-H^+^-ATPase (1:10,000, Agrisera, Vännäs, Sweden, AS07 260), and anti-PsbA (1:20,000, Agrisera, Vännäs, Sweden, AS05084).

### 2.7. ATP Hydrolysis Assays

Activity of the PM- and VM-H^+^-ATPases was measured as Pi release from ATP (Sigma-Aldrich, St. Louis, MO, USA) as described in [22] in the presence of specific inhibitors: Na_3_VO_4_ (Sigma-Aldrich, St. Louis, MO, USA), a specific inhibitor of the plasma membrane H^+^-ATPase, or KNO_3_ (Sigma-Aldrich, St. Louis, MO, USA) as an inhibitor of the tonoplast ATPase. Phosphate was measured as the reduced phosphomolybdate complex by a colorimetric method.

### 2.8. Membrane and Probe Models

The theoretical membrane was constructed using the CHARMM-GUI interface [26] with membrane builder. The composition was set to 10% β-Sitosterol, 35% DPPC (dipalmitoylphosphatidylcholine), 35% DOPC (dioleoylphosphatidylcholine), and 20% Cer 24:0 (ceramide d18:1/24:0). KCl was added to neutralize the system and SPC as water molecules. DPH, TMA-DPH, and cis-PA were simulated using SwissParam website [27]. Assembled systems were visualized using Open-Source PyMOL (The PyMOL Molecular Graphics System, Version 1.3, Schrödinger, LLC). 

### 2.9. Segment Analysis of Arrhenius Plots

Analysis of segments was performed in R (version 4.0.3, R Core Team, 2020, Vienna, Austria) using the piecewise linear segmentation of ordered data by a dynamic programming algorithm “dpseg” (R package Version 0.1.1) with a breakpoint penalty of 1 × 10^−4^, minl = 3 and jumps = 0. 

### 2.10. Statistics

Data are presented as the mean ± SEM and compared by Student’s t-test (unpaired) or ANOVA using GraphPad InStat version 3.10 (GraphPad Software, San Diego, CA, USA).

## 3. Results

### 3.1. Purity Assessment of Total, Vacuolar, and Plasma Membrane Vesicles Isolated from Arabidopsis Thaliana

**Figure 1:**
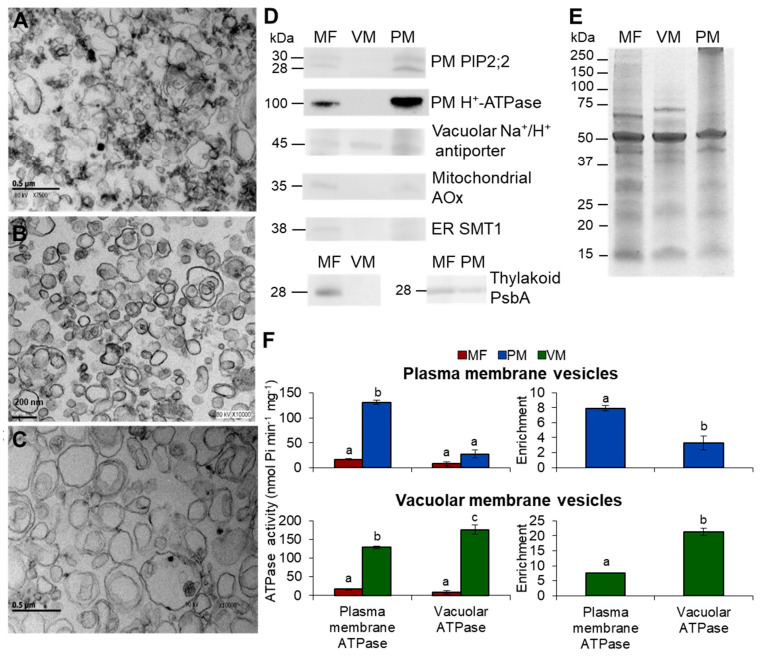
Purity assessment of isolated membranes from *Arabidopsis thaliana* leaves. Transmission electron micrographs from microsomal fraction (MF) isolated by differential centrifugation (**A**), plasma membrane (PM) purified by two-aqueous polymer partitioning system (**B**), and vacuolar membrane (VM) isolated by free flow zonal electrophoresis (FFZE) (**C**) from *Arabidopsis thaliana* leaves. (**D**) Immunodetection of membrane markers. MF, VM, and PM proteins were separated by SDS-PAGE and detected by immunoblot with specific antibodies as described under Materials and Methods. The H^+^-ATPase and the aquaporins PIP2;2 are the PM markers. The Na^+^/H^+^ antiporter is the VM marker. The alternative oxidase AOx is the mitochondrial inner membrane marker. The sterol methyltransferase SMT1 is the ER marker. The PsbA protein is the thylakoid marker. (**E**) Replicate gel from the immunoblotted proteins stained with Coomassie blue. (**F**) Determination of ATP hydrolysis from PM H^+^-ATPase and VM H^+^-ATPase in the PM and VM vesicles. ATPase activity was measured as Pi release from ATP as described under Materials and Methods. Ultrastructure analysis consisted of 3 replicates from three preparations independently obtained. Western blots experiments were performed using 2 to 5 independent membrane preparations with 2 to 6 technical replicates. ATP hydrolysis assays included 5 independent MF preparations and 5 replicates, 3 independent VM preparations and 3 replicates, 6 independent PM preparations, and 15 replicates. Values are expressed as means ± s.e. from 6 to 15 replicates. The different letters a, b, and c indicate statistically significant difference at *p* < 0.05 (ANOVA test).

To study membrane fluidity, we used three preparations: MF, which contained vesicles from all membrane compartments, vacuolar membrane (VM), and plasma membrane (PM). The purity of these preparations was assessed in terms of ultrastructure and the presence of membrane markers (Figure 1).

Ultrastructural analysis of the MF (Figure 1A) showed a heterogeneous mixture of vesicles of different sizes and shapes, with some amorphous, heavily stained material. In contrast, the PM (Figure 1B) and VM (Figure 1C) preparations revealed vesicle populations of homogeneous shape and size lacking unstructured material. Vesicle enrichment was assessed by immunodetection of membrane protein markers (Figure 1D,E). All membrane markers were clearly represented in the MF fraction. As expected, the PM preparation was enriched in the PM markers (H^+^-ATPase and the aquaporins PIP2;2), while the VM marker (vacuolar Na^+^/H^+^ antiporter), the mitochondrial marker (alternative oxidase), was poorly represented; the ER marker (sterol methyltransferase 1) was absent, and the thylakoid membrane marker (PsbA protein) was clearly present. The opposite was found in the VM preparation, which showed enrichment in the vacuolar translocator and non-detected levels of the other five membrane markers (Figure 1D). In order to gain more information about the enrichment of the PM and VM vesicles, we measured the ATPase activity from the PM H^+^-ATPase and from the VM H^+^-ATPase in both membrane preparations (Figure 1F). A good enrichment of both membranes was observed according to these activities: 20-fold for VM and 8-fold for the PM as compared to their respective MFs. Altogether, the global assessment of the three purification criteria: ultrastructure, membrane markers, and activity markers, indicated a substantial enrichment of the PM and VM preparations.

### 3.2. PM, but Not VM or MF, Show the Highest Fluidity Values and Responds to External Temperature Variations

**Figure 2:**
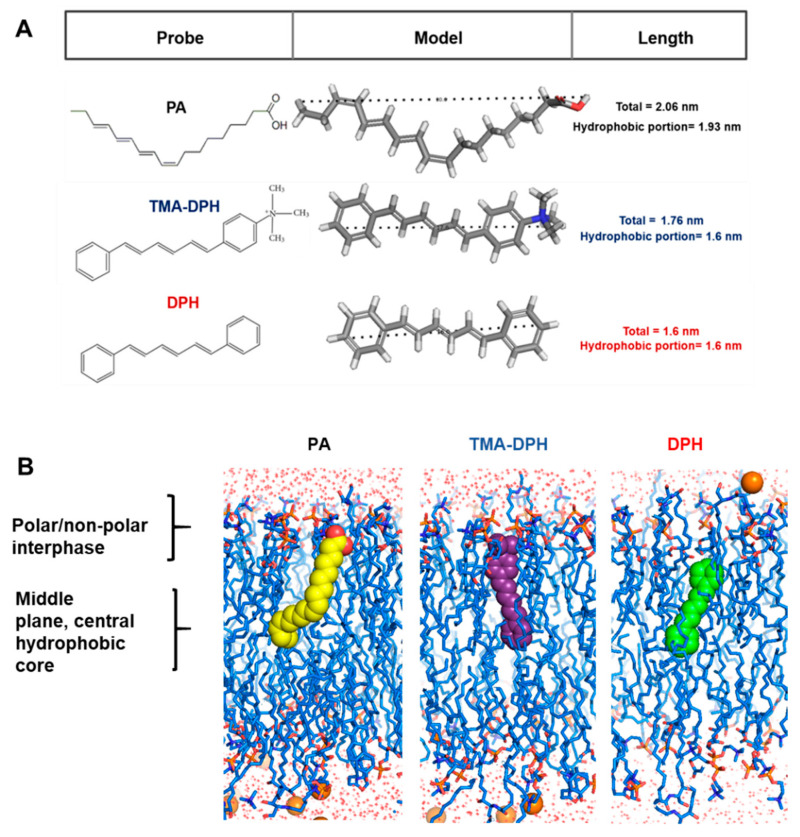
Structural dimensions and membrane localization of the membrane fluorescent probes used in this study. (**A**) Chemical structures and dimensions of the probes. PA is a conjugated polyunsaturated fatty acid that preferentially partitions into liquid disordered domains in membranes and covers the entire length of a fatty acid chain of 18C. TMA-DPH is derived from DPH and contains a cationic trimethylammonium group that functions as an anchor to the membrane surface; it can reach 1.6 nm into one monolayer. DPH inserts into the most hydrophobic portion of a lipid bilayer and has a longitude of 1.6 nm. (**B**) Membrane bilayer (blue) and molecular models of the fluorescent probes inserted are based on molecular dynamics simulations in the literature.

To study whether the isolated membranes were thermoresponsive, we analyzed fluidity as a function of temperature. For each sample, temperature was increased and decreased from −5 to 41 °C in succession at a rate of 2 °C every 2 min. We determined membrane fluidity by measuring FP with three different probes DPH, TMA-DPH, and PA (Figure 2). These probes were selected because of their differential ability to report the state of order from distinct regions of the bilayer according to their chemical structure (Figure 2A,B) [28,29]. DPH is a highly hydrophobic molecule, planar and symmetrical, which avoids the polar region of the bilayer (Figure 2B). For lateral allocation, DPH partitions into the liquid ordered and liquid disordered phases and is capable of fast, long-range lateral motion [30,31]. Theoretically, the TMA-DPH probe exhibits no preference for any particular phase [30] but contains a positively charged trimethylammonium that acts as a surface anchor that restricts large-range lateral displacements (Figure 2B) [31]. *cis*-PA is a highly unsaturated fatty acid (FA) with a natural conjugated tetraene located between C9 and C16 from the acyl chain and a polar moiety that anchors the FA to the surface of the lipid bilayer. Since PA is excluded from the liquid ordered phases due to its highly unsaturated character (Figure 2B), it is mainly used to monitor membrane fluidity in liquid disordered phase regions [29,32].

**Figure 3:**
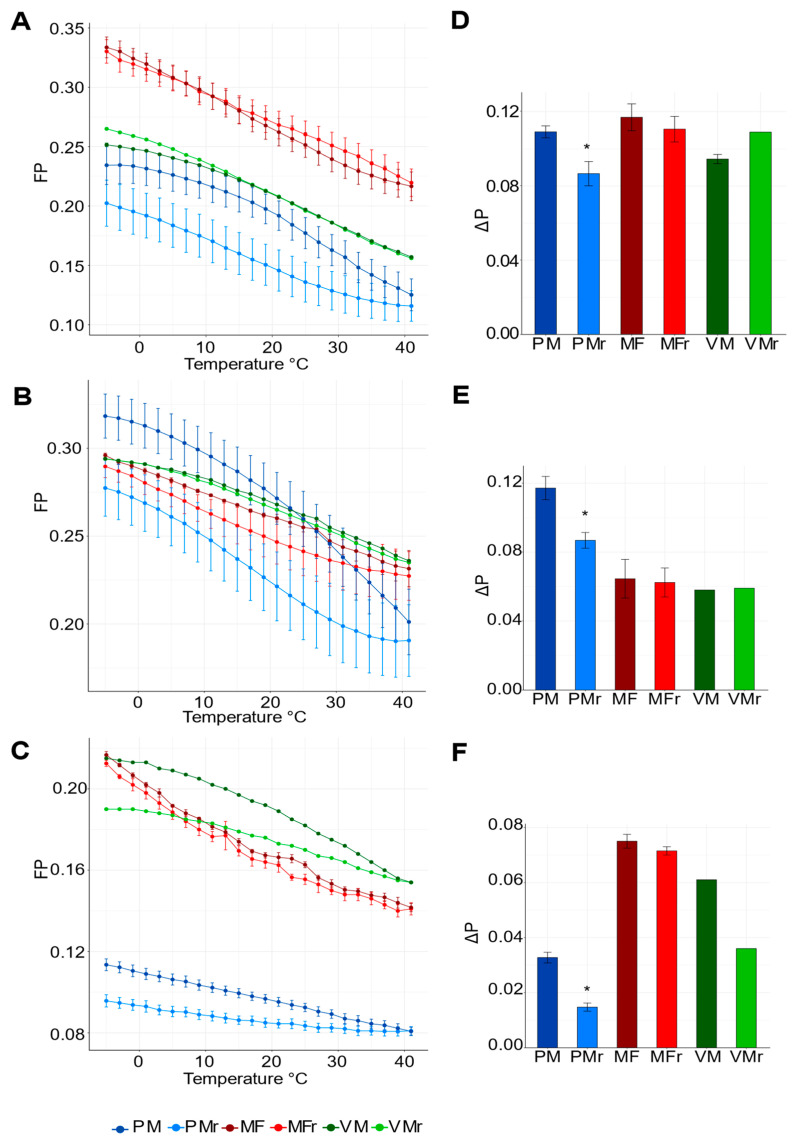
Effect of temperature on membrane fluidity (monitored as fluorescence polarization, FP) of microsomal fraction (MF), vacuolar membrane (VM), and isolated plasma membrane (PM). FP profile of MF (red, mean ± SEM n = 4), VM (green, mean ± SEM n = 2) and isolated PM (blue, mean ± SEM n = 5) from wild type plants using (**A**) DPH, (**B**) TMA-DPH, or (**C**) PA as membrane fluidity probes. Dark color represents data collected while warming membranes (MF, VM, PM from −5 to 41 °C) and the light color for cooling membranes (MFr, VMr, PMr from 41 to −5 °C). Total difference of FP, ΔP values (final FP value – first FP value of every curve shown on the left side panel), of MF, VM, and PM using (**D**) DPH, (**E**) TMA-DPH, or (**F**) PA. Statistical differences were calculated using a two-tailed t-test comparing the cooling and heating conditions, * *p* < 0.05. See Materials and Methods for details on the FP set up.

For all probes, FP values were higher at low temperatures (implying a less fluid membrane) and lower at high temperatures (indicating a more fluid membrane) (Figure 3). This behavior was independent of the probe and membrane source used, indicating that our conditions and measurements were correct and reliable [29,30]. 

Analysis of FP in the −5 to 41 °C direction using DPH showed that MF had the highest absolute values of FP, indicating that MF had a low average fluidity (Figure 3A). In the same analysis, the VM were more fluid than MF, whereas PM showed the lowest FP values and accordingly, the highest fluidity states at all temperatures tested (Figure 3A). The FP profile with DPH indicated that PM was more fluid in general and had the lowest polarization values when gradually cooling compared to MF and VM, which had the same values in either direction of temperature change (Figure 3A,D). Interestingly, when comparing the cooling and warming line trajectories, the distance between them was greater in PM with the three probes tested. This value suggested a wide response of PF to the total temperature interval. VM had a similar pattern only with the PA probe (Figure 3A–C).

There was little difference in polarization measurements for PM, MF, and VM when using the TMA-DPH probe (Figure 3B). We reasoned that instead of reporting the degree of order, as shown in Figure 3A, TMA-DPH was imposing order (Figure 3B) and consequently constrained movement due to interactions of the trimethylammonium group with the polar region of lipids. Measurements of FP with PA showed, as with DPH, that PM was more fluid than MF and VM (Figure 3C). 

To estimate the capacity of every membrane to recover the initial values of fluidity once it had been subjected to successive increases or decreases in temperature, the fluidity values reached at the highest and lowest temperatures were used to calculate ΔP values for both directions, warming or cooling (Figure 3D–F). ΔP values were significantly different between cooling and warming for PM; however, MF had no significant difference between the final and initial measurements and VM statistical analysis was not possible to perform due to the low number of biological preparations. This pattern was observed with the three probes in the three types of membrane preparations. 

**Figure 4:**
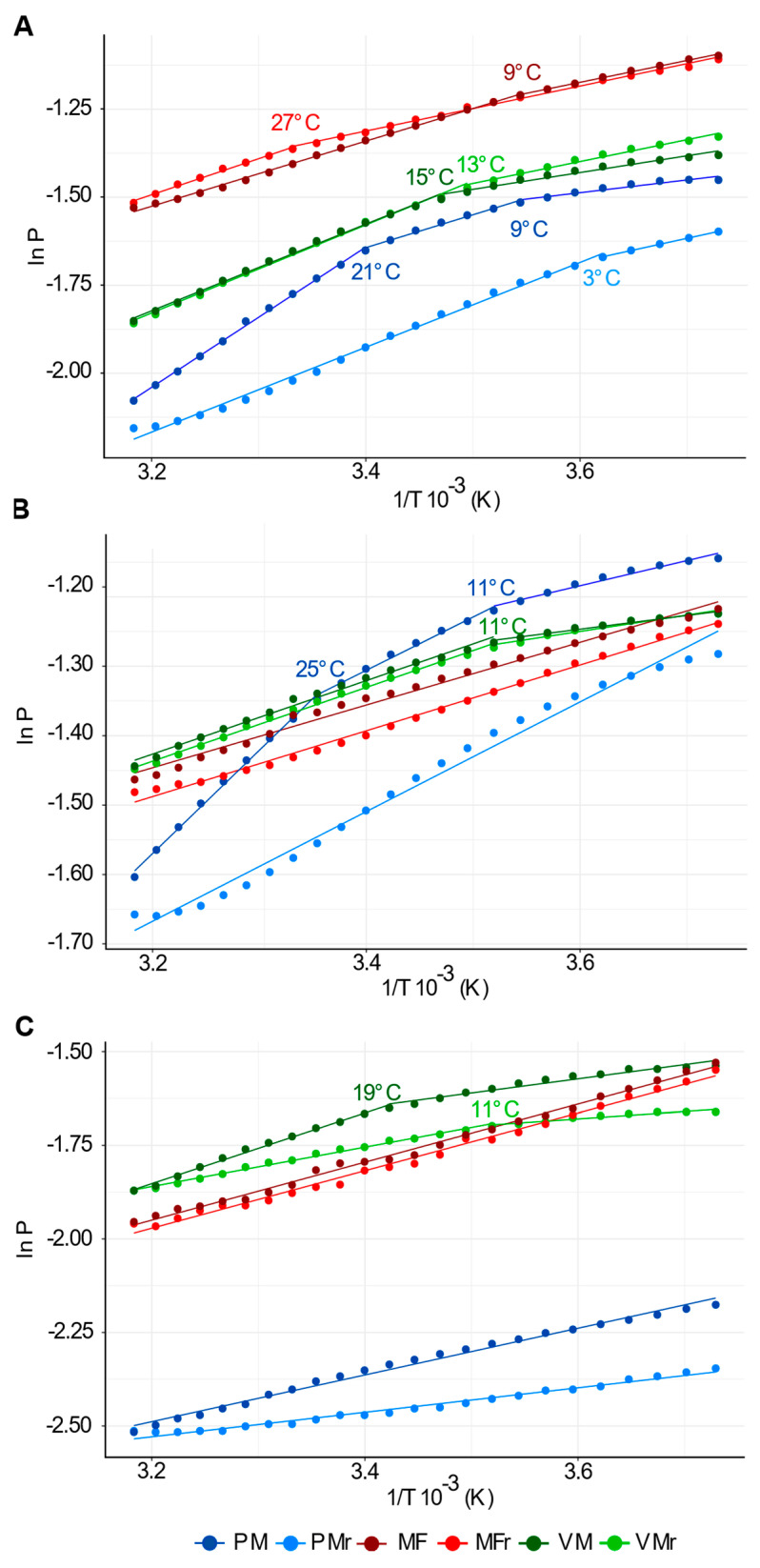
Arrhenius plots of the FP values from Figure 3. Data are shown as natural logarithm (ln) of P (polarization of fluorescence) versus the inverse temperature in Kelvin degrees (1/T 10^−3^) using (**A**) DPH, (**B**) TMA-DPH, or (**C**) PA as membrane fluidity probes. Curves were then analyzed by piecewise regression to find segments and breakpoints using “dpseg” package in R. Different segments and the temperature at which the breaking points occur are shown. Colors of dots and numbers at the breaking points of the lines correspond to the type of membrane and the direction of temperature change as indicated in the bottom labels. MF, VM, PM are determinations from membrane preparations subjected to a gradual increase of temperature. MFr, VMr, PMr are determinations from membrane preparations subjected to a gradual decrease of temperature.

To determine whether the polarization of the fluorescent probes could reveal transition phases, Arrhenius plots (Ln P vs 1/T 10^−3^) from the FP data (Figure 3) were calculated (Figure 4) [33,34]. At a phase transition temperature, membrane lipid configuration shifts from one state of order and lateral packing to another one (mesomorphic phase states). This is denoted as curved, non-linear plots that can be decomposed into straight lines with different slopes. Piecewise linear segmentation analysis was used to detect the number of breaking points and the segments.

PMs showed a curved relationship in the Arrhenius plots when using DPH and TMA-DPH (Figure 4A,B) and was the only system to have three segments and two breaking points, which only occurred during warming. The transition temperatures were 9 and 21 °C with DPH and 11 and 25 °C with TMA-DPH. After warming, membranes were then cooled back to −5 °C, which removed the breaking points and made changes in fluidity directly proportional to temperature. Regarding VM and MF, both were characterized by one breaking point for warming and cooling using DPH while there were inflexions in VM in both temperature directions when using TMA-DPH (Figure 4A,B). Contrary to MF and VM that have almost identical lines for cooling and heating, PMs showed irreversible changes in fluidity, which can be interpreted as thermal hysteresis. 

Data from PA plots showed straight lines for PM and MF, and one breaking point for VM per change in temperature (Figure 4C). This contrasts with the PM curved lines seen before.

This work provides experimental evidence on the mechanism by which external temperature variation can rapidly impact the fluidity state of the PM across a broad temperature range without metabolic energy cost. 

## 4. Discussion

### 4.1. PM Exhibits High Fluidity, Phase Transitions, and Irreversible Behavior during a Heating/Cooling Cycle

We found that PM was highly fluid compared to MF and VM, during both heating and cooling across 23 consecutive measurements in a temperature range from −5 to 41 °C. This was clearly observed with DPH and PA, reporters of the lipid hydrophobic tails environment. Besides lower fluidity, MF and VM showed lines with none or one phase transitions during heating or cooling in the presence of DPH, TMA-DPH, or PA. These results are in agreement with previous reports, in which VM isolated from *Kalanchoë daigremontiana* subjected to a gradual rise in temperature from −5 to 40 °C showed an increase in fluidity measured with DPH or β-py-C10-HPC, although no changes in slope were observed [35]. Similar thermal screenings in thylakoid and grana preparations showed that FP curves of DPH lacked phase transitions [36]. In contrast, we found that PM displayed two phase transitions around 9 and 21 °C when using DPH in a temperature range spanning from −5 to 41 °C. In previous studies, changes in slope were also found in PM isolated from maize roots in FP curves with DPH [37] and a phase transition temperature of −3 °C was also reported in PM from orchard grass, however, this shift depended on the membrane composition which varied in response to seasonal temperatures [38]. Our results with DPH show that when cooled, PM had a Tm of 3 °C, which highlights the importance of the direction of temperature change (Figure 4). The detection of phase transitions in the PM by FP of DPH and TMA-DPH revealed the capacity of the membrane to largely modify its supramolecular order in response to temperature changes. In addition, the decreasing or increasing values of FP every 2 °C in all the points along the curves suggests that the membrane fluidity responded to discrete and small changes of temperature in ambient ranges.

To our knowledge, although thermal screening has been performed in plant PM [37,38,39,40,41], no thermal screening of membrane stability (carried out by sequential warming and cooling) using purified plant membranes has been reported. We found that, while MF and VM had fully reversible membrane fluidity states, the fluidity of PM recorded during a gradual rise of temperature followed a trajectory that was not quantitatively reversed when a subsequent gradual decrease in temperature was imposed (Figure 3). This physical property of hysteresis in the PM, defined as the dependence of the state of a system on its previous history, is present in systems exhibiting phase transitions, largely regarded as thermodynamically irreversible changes [42]. Using high-sensitivity differential scanning calorimetry and X-ray diffraction, Holopainen et al. (2000) used model membranes to show that ceramides cause hysteresis in thermal phase behavior [43]. In addition, protein mediators of hysteresis have been identified in plants, which have been localized in PM from *Solanum dulcamara* [44]. Hysteresis can be a physiologically relevant process in plants upon unexpected thermal changes, a common natural condition for plants, as it would allow the PM to retain a record of previous temperature changes. This constitutes an advantageous property for organisms that are constantly exposed to thermal variations. However, it is also conceivable that some other non-hysteresis-related mechanism, which may be associated with membrane lability, could be responsible for the observed, non-reversible effect. Still, it was remarkable that such behavior of membrane fluidity was exclusively observed in the PM and not in the MF or the VM. 

### 4.2. Identification of a Temperature Sensor in Plants

A central issue in the study of the molecular elucidation of temperature responses in plants has been the identification of the sensor, receptor, or detector of temperature, since this is the departing point of any mechanism that includes a response. Therefore, several candidates have been proposed.

Perception of thermal fluctuations throughout the evolutionary scale involves DNA and RNA sensors as well as proteins and the membrane lipid phase. DesK in *Bacillus subtilis* is activated by a conformational change triggered by an increase in membrane thickness in response to cold sensing [45,46]. In the cyanobacteria *Synechocystis*, Hik33 is a cold sensor that dimerizes upon one of its domains detecting changes in membrane fluidity [47,48]. In both cases, the cold sensor undergoes autophosphorylation, leading to the expression of a FA desaturase, which increases membrane fluidity as a final response [8]. However, what these proteins sense is a change in membrane fluidity, thereby stressing the importance of the PM as a crucial primary thermal indicator.

In plants, cold, more than heat responses, have been studied at the molecular level [49,50]. Early events upon cold exposure have previously been identified, including an increase in cytosolic calcium concentration [51], cytoskeleton disassembly [52,53], PLC activation [54], and MAP kinase activation [55]. However, all these early events occur downstream of an initial membrane stiffening transition, which represents a departing point for a signaling pathway in response to cold exposure.

As to heat perception, it is recognized that early events include changes in calcium fluxes and heat shock protein expression levels, nevertheless a calcium channel proposed as a thermosensor is yet to be identified [56,57,58]. Molecules, such as phototropin, the clock component ELF3, and secondary RNA structures, have been proposed as thermosensors [11,59] but only Phytochrome B has been acknowledged as a photothermal receptor and only for mildly high temperatures [11,59,60]. The chloroplast has also been implied in sensory activities of environmental stresses, including gene expression of the HSP70 proteins [61]. Furthermore, photosynthetic components as the plastoquinone pool, tocopherols or intermediates of chlorophyll synthesis have been postulated as chloroplast sensors of light/heat because of the sensitivity of the photosynthetic apparatus to high temperatures [62,63,64].

### 4.3. The Support for the Hypothesis of the PM Fluidity as a Temperature Detector

Earlier and recent reviews have agreed on the absence of a defined plant temperature receptor in plants. However, all authors have acknowledged the feasibility of the hypothesis of the PM fluidity as the primary sensor of temperature. They have considered the PM’s advantageous properties: mesomorphic behavior and capacity of containing/generating signaling transducers (kinases, lipids, Ca^2+^ fluxes) [5,8,49,61,65,66]. However, the hypothesis of the PM fluidity as a temperature detector has not been specifically addressed in experimental terms in recent times. We approached this problem using isolated membranes from different cellular sources and compared their fluidity behavior at different depths of the membrane. Our results indicate that the PM fluidity is sensitive to small changes in temperature and responds rapidly to these temperature variations, and it can display hysteresis and it varies in response to a temperature interval to which plants are exposed in their natural habitats. Moreover, the PM is a dynamic but stable cell structure whose fluidity depends at great extent of the lipid mesomorphism, a phenomenon that does not utilize the metabolic energy of the cell. Therefore, our results support the hypothesis of PM fluidity as a broad-spectrum thermal detector in plants. This detector contains the appropriate features, such as kinases and other components, that may generate second messengers, such as ion fluxes or lipids. These components may transfer the information of the changes in membrane lipid conformation through diverse downstream signaling pathways that may translate the thermal signals to specific metabolic responses. A link between changes in the PM fluidity in response to changes in temperature and a final adjustment in fatty acid desaturation has been described [13]. The signaling components immediately connected to the changes in membrane fluidity and the subsequent elements remain to be identified. The absence of evidence is not evidence of absence.

Interestingly, homeothermic organisms possessing a constant internal temperature are equipped with thermal sensor proteins, typically ionic channels, specific to certain temperature intervals, whose activity may be influenced by specific bound lipids but not by the behavior of membrane bulk lipids [45,67]. It is therefore reasonable to propose that poikilothermic organisms took advantage of an inherent property of their membranes for temperature sensing, namely their PM lipid mesomorphism.

## Data Availability

The data presented in this study are available on request from the corresponding author.
